# Hereditary neurodegenerative disorders with spastic-ataxic manifestations: genetic and clinical insights into previously reported variants

**DOI:** 10.1007/s10048-026-00929-9

**Published:** 2026-08-31

**Authors:** Riaz Ahmad, Kanwal Ayaz, Misbah Naeem Khan, Muhammad Naeem, Henry Houlden

**Affiliations:** 1 Medical Genetics Research Laboratory, Department of Biotechnology, Quaid-i-Azam University, Islamabad, 45320 Pakistan; 2 Department of Neuromuscular Disorders, UCL Queen Square Institute of Neurology, Queen Square House, London, WC1N 3BG UK

**Keywords:** Ataxia, Hereditary neurodegenerative disorders, Next-generation sequencing, Spastic paraplegia

## Abstract

Hereditary neurodegenerative disorders comprise a heterogeneous group of genetic conditions that affect the central and/or peripheral nervous system, often presenting with hallmark clinical features including ataxia, spasticity, dystonia and neuropathy. Our study aims to investigate the underlying cause of rare and overlapping phenotypes of inherited neurodegenerative disorders in the four familial cases of the Pakistani population. Four Pakistani families with a wide range of spastic-ataxic features were evaluated using exome sequencing, homozygosity mapping, and Sanger sequencing. These molecular studies identified four known pathogenic variants: *CYP2U1*:c.604G > A (p.Glu202Lys), *ATXN1* expansion: [(CAG)46/(CAG)27], *ATM*:c.103 C > T (p.Arg35*), and *COQ4*:c.577 C > T (p.Pro193Ser). All these phenotypes were correlated with the literature-based reported cases of rare neurological disorders. We present previously reported pathogenic variants in our enrolled 13 affected individuals from four unrelated families. Our findings underscore the pathogenic relevance of the identified variants and pinpoint their importance for regional diagnostic and genetic counseling strategies.

## Background

Hereditary neurodegenerative conditions represent a diverse group of inherited diseases characterized by progressive dysfunction and degeneration of the central and/or peripheral nervous system. These diseases arise from pathogenic mutations in genes essential for neuronal development, maintenance, and survival, leading to a gradual loss of motor and cognitive functions. Clinically, they manifest with overlapping symptoms including ataxia, spasticity, dystonia, and peripheral neuropathy, reflecting the diverse yet interconnected pathogenic mechanisms affecting motor and cerebellar pathways. The extensive genetic and clinical heterogeneity observed in these disorders complicates diagnosis and underscores the need for in-depth molecular investigations to delineate precise genotype-phenotype relationships [1]. 

Spinocerebellar disorders or hereditary spinocerebellar degenerations are a group of genetically and clinically heterogeneous neurodegenerative disorders, including spastic paraplegias and inherited cerebellar ataxias. Cerebellar ataxias encompass a movement or gait disorder caused by the progressive dysfunction of the cerebellum that leads to problems with walking and balance, resulting in significant disability [2]. 

Hereditary spastic paraplegias (HSP) are also a clinico-genetically diverse group of neurodegenerative conditions because of retrograde axonal degeneration of the dorsal column and corticospinal tract, causing spasticity of lower limb extremities and hyperreflexia [3]. The following are the overlapping non-neurological and neurological clinical manifestations of ataxias and spastic paraplegias: dysarthria, spasticity, spastic paraparesis, dystonia, dysphagia, cognitive impairment, and ocular abnormalities [4]. 

The prevalence rate of spinocerebellar degeneration disorders is approximately 1:10,000 individuals globally; thus, these are considerably rare disorders. About 70% of these rare diseases are inherited, and most of them affect the nervous system [5]. NGS is commonly employed for the diagnosis of Mendelian disorders, comprising neurological conditions [6]. Recent advances in NGS-based techniques have substantially improved the identification of genes associated with spinocerebellar degeneration disorders, enhancing diagnostic yield and expanding the understanding of their genetic heterogeneity [4]. The advent of NGS, which is effective, cheap, and even more available, permits the detection of likely pathogenic (LP), pathogenic (P), and even variants of unknown significance (VUS). Furthermore, NGS can be employed early for such patients before detailed laboratory investigations. The initial genotyping approach, which is cost-effective and more pragmatic, requires collaboration between clinicians and geneticists to interpret results in relation to phenotypes. Nonetheless, proper clinical examination - which excludes acquired factors, precisely defines the phenotype, and ensures patient follow-up - is strongly advised [7]. 

In the current study, we investigated four unrelated Pakistani families affected with diverse manifestations of spastic-ataxic-related disorders. From a large cohort of Pakistani families, we have identified these genetically confirmed cases presenting with spastic-ataxic and overlapping neurodegenerative phenotypes. Our presented work highlights the significance of known pathogenic variants as valuable markers for diagnosis, counselling, and preventive strategies in the local population.

## Materials and methods

This study was conducted in accordance with the principles of the Declaration of Helsinki. Ethical approval was obtained from the respective Institutional Review Board. Informed consent was obtained from all adult participants and from parents or legal guardians for minors.

**Fig. 1:**
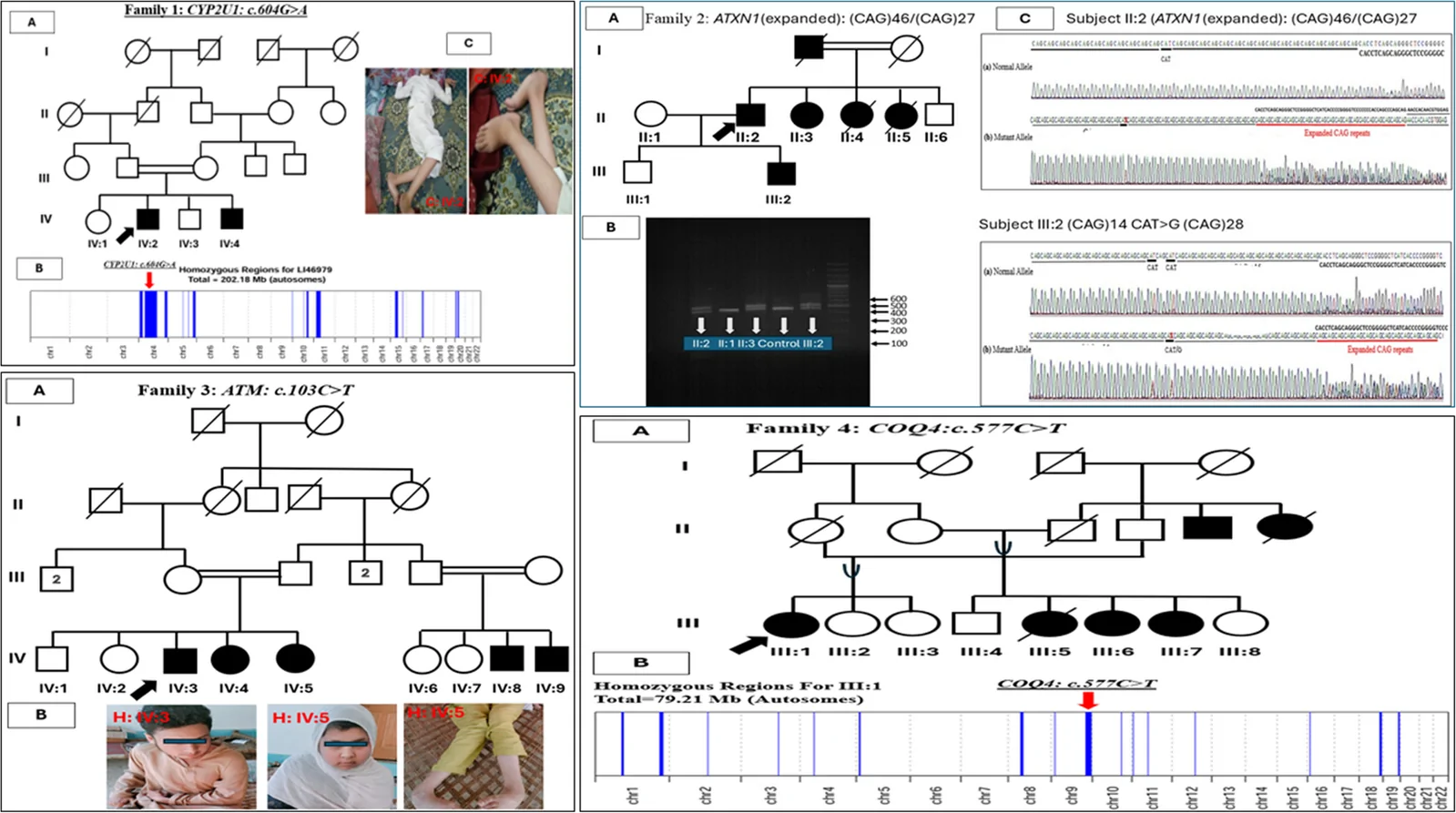
Family 1 **A** Four-generation pedigree: circles represent females while squares denote males; filled shapes were used to differentiate healthy and affected individuals; an arrow was used for the proband, and double lines represent consanguinity. **B** Homozygosity mapping: The 4th chromosome showed a high homozygous region. **C** Clinical photographs of the affected individual (IV:2). Family 2 (**A**) Three-generation pedigree. **B** Agarose gel-based identification of heterozygous and homozygous bands. **C** DNA sequencing electropherograms showing *ATXN1* CAG repeats in the affected subjects, II:2 and III: 2. Family 3 (**A**) Four-generation pedigree. **B** Clinical representation of patients (IV:3 & IV:5). Family 4 (**A**) Three-generation pedigree. **B** Homozygosity mapping of proband (III:1)

Peripheral blood samples were obtained from all affected individuals after informed consent. Genomic DNA was extracted from collected samples through the Qiagen DNA extraction kit (Cat# 56,304, QIAamp, Qiagen, Valencia, CA, USA). Extracted genomic DNA was further quantified through a Quantus Fluorometer (Cat# E6150, Promega, Madison, WI, USA). Exome sequencing was performed (probands and available family members of all families) using the Agilent SureSelect Human All Exome V6 Kit (Agilent Technologies, Santa Clara, CA, USA) as described previously [8]. High-throughput sequencing was performed (for probands of all families; Fig. 1) via Illumina NovaSeq 6000 (Illumina, Santa Clara, CA, USA) with paired-end sequencing (PE150). The retrieved sequencing reads were further aligned to the reference genome via Burrows-Wheeler Aligner v0.7.17 (http://bio-bwa.sourceforge.net/). For the enrolled probands, the hg38 assembly was chosen overall for sequencing and filtration. The obtained BAM files were sorted using SAMtools (v1.8) and duplicated reads were identified through Picard (v2.18.9). Genome Analysis Toolkit (GATK; http://www.broadinstitute.org/gatk/) v4.0 was used for genotyping. Functional annotation was done by ANNOVAR (http://www.openbioinformatics.org/annovar/) and filtration with the help of FILTUS. Disease-causing or pathogenic variants were selected according to our previously used filtration strategy, as available in Figure S1 [23]. WES data were filtered based on standard quality metrics, including a minimum read depth of ≥ 20×, variant quality score ≥ 30, and minor allele frequency < 1% in population databases. Only high-confidence variants supported by sufficient read depth and quality scores were retained for downstream analysis. For the probands of family 1 and 4, homozygosity mapping was used with the default setting of the AutoMap tool.

Different in silico tools (SIFT, CADD score, and Polyphen-2) were used to predict functional effects of the filtered variants. ACMG guidelines and the latest literature were considered to classify identified variants.

The Primer3Web (version 4.1.0) tool was used to design primers (Forward primer: GGTCCTCCCAATACAGTGGA, Reverse primer: GTGTGTGGGATCATCGTCTG) for the targeted sequence amplification of the *ATXN1* gene. PCR followed by agarose gel electrophoresis was performed to visualize the CAG repeat expansion in family 2. Targeted PCR products with double bands (heterozygous) were separated by excising the respective bands from 2% agarose gel alongside a 100-base pair DNA ladder (Thermo Scientific USA) and purified using the GeneJET Gel extraction kit (Thermo Scientific, USA). The separated normal and mutant alleles were subjected to Sanger sequencing using an ABI-3730 Genetic Analyzer. Sanger sequencing was used to confirm the segregation of the variant in the family. BioEdit Sequence Alignment Editor (Version 7.2.6) tool was used to visualize and analyze the sequence files generated by the genetic analyzer.

## Results

Clinical findings of all the affected individuals are summarized in Table 1, with clinical photographs shown in Fig. 1 and their pedigrees presented in Fig. 1.

**Table 1:** A summarized form of clinical details of four unrelated Pakistani families (1–4)

ID	Patient ID	Gender	Age	Onset	Likely OMIM syndrome	Clinical symptoms
Family-1	IV:2 IV:4	M M	14 11	2 years 1 year	Spastic paraplegia 56, autosomal recessive, OMIM: 615,030	Spastic muscles, difficulty in speech, inability to ambulate, weak neck holding, hyperreflexia, deformed limbs, severe intellectual disability, muscle atrophy, and no ocular anomalies. **Interfamilial heterogeneity**: All symptoms are the same; however, patient IV:2 exhibited an earlier onset compared to IV:4 and retained limited crawling ability. Patient IV:2 presented with hyperreflexia predominantly affecting the lower limbs, while patient IV:4 exhibited hyperreflexia in the upper limbs. **Estimated SPRS range**: Based on clinical severity, estimated Spastic Paraplegia Rating Scale (SPRS) scores ranged from approximately 40–45 in patient IV:2 to 45–50 in patient IV:4, reflecting very severe disease with near-complete loss of motor independence.
Family-2	II:2 II:3 II:4 II:5 III:2	M F F F M	48 NA NA NA 21	26 NA NA NA 17	Spinocerebellar Ataxia Type 1, OMIM:164,400	Cerebellar ataxia, dysarthria, difficulty during gait, progressive weakness in both limbs, cerebellar signs, cognitive decline, motor neuropathy, cerebellar atrophy, generalized brain atrophy, moderate dilation of CSF spaces, and prominent cerebellar folia. **Interfamilial heterogeneity**: Patient (III:2) presented with neurological complications much earlier, experiencing seizures and stroke during infancy, followed by intellectual disability, suggesting a more severe or atypical clinical course. Based on clinical severity, estimated SARA scores ranged from approximately 28–34 in typical affected individuals to 34–40 in patient III:2, while corresponding ICARS estimates ranged from 70–85 to 85–100, respectively, indicating severe to very severe cerebellar dysfunction.
Family-3	IV:3 IV:4 IV:5 IV:8 IV:9	M F F M M	17 14 12 13 11	1 year 2 years 1years By birth 1 year	Ataxia-telangiectasia, OMIM:208,900	Cerebellar ataxia, slurred speech, intellectual disabilities, dysarthric speech, dystonia, tremors, seizures, oculomotor abnormalities, short stature, bronchitis, telangiectasia, conjunctival delayed puberty, and strabismus. **Interfamilial heterogeneity**: All mentioned signs were observed, but IV:3 has more prominent cutaneous telangiectasia. Bronchitis was frequently observed in IV:4.
Family-4	III:1	F	17	1 year	Spastic ataxia 10, Autosomal recessive, OMIM: 620,666	Seizures, walking difficulties, abnormal gait, spastic paraplegia, lower limbs, spasticity, dysarthria, hyperreflexia and intellectual disabilities. **Interfamilial heterogeneity: NA**

### Family 1

Subject (IV:2) is a 14-year-old boy, the second of four children born to healthy consanguineous parents. Perinatal complications and growth constraints were normal at the first year of life, and then disease onset occurred at the age of 2 years. Metabolic and biochemical markers were within the normal range. Clinical signs in subjects (IV:2; IV:4) were spastic muscles, difficulty in speech, unable to move and stand, weak neck holding, deformed both limbs, and toe walking. No digestive or respiratory complications were observed in either patient. Details of interfamilial heterogeneity are provided in Table 1. Clinical signs are matched with spastic paraplegia 56, autosomal recessive (OMIM: 615030).

### Family 2

The pedigree analysis of family 2 revealed an autosomal dominant pattern, with four affected subjects in the second generation (Fig. 2). Co-segregation was confirmed through gel and Sanger sequencing. The proband (II:2) was 48 years old, and the onset occurred at 26 years of age. Clinical features of the proband were initially suspected to be of spinocerebellar ataxia type 1 running in the family. All patients exhibited cerebellar ataxia, dysarthria, gait difficulty, and progressive weakness in both limbs (Table 1). The proband’s physical examination showed cerebellar signs, cognitive decline, and motor neuropathy. MRI report showed cerebellar atrophy and generalized brain atrophy consistent with spinocerebellar ataxia. In addition, MRI showed moderate dilation of both extra- and intra-CSF spaces and prominent cerebellar folia. The size of the medulla and pons, and even the spinal cord is reduced.

### Family 3

Family 3 includes five affected individuals born to consanguineous parents. The disease onset occurs at 1 to 2 years of life. Clinical features were the same in all patients, including cerebellar ataxia, dysarthric speech, dystonia, tremor, seizures, and oculomotor abnormalities. Physical examination showed short stature, conjunctival telangiectasia, and strabismus. Minor clinical heterogeneity was observed in all affected individuals, and details are provided in Table 1. All these patients were less responsive to their surroundings at the infancy stage. For self-care and feeding, patients are dependent on others, and walking and running were never achieved. Clinically, the phenotypes of our cohort were matched with ataxia telangiectasia (OMIM: 208900).

### Family 4

Family 4 was recruited with six patients (Fig. 2). The proband was a 17-year-old, born with normal pregnancy. At the age of 1 year, she experienced her first seizure. In early childhood, she presented with delayed sitting and walking problems. In addition, her symptoms worsened with dysarthria, hyperreflexia, developmental delay, walking difficulties, abnormal gait, spastic paraplegia, lower limb spasticity, and intellectual disabilities. She had a family history of the same disease, and all affected individuals presented with similar symptoms.

The annotations, minor allelic frequencies (MAF), and in silico prediction tools for the identified variants in the selected families are enlisted in Table 2. In family 1, the already cited missense (c.604G > A; p.Glu202Lys) variant [9] in the *CYP2U1* was diagnosed. In the *ATXN1* gene, previously known CAG expansion (more than 43 repeats) was identified in family 2 with a heterozygous pattern and classified as pathogenic (P). In families 3 and 4, two previously reported variants were identified: c.103 C > T; p.Arg35Ter in the *ATM* gene [10] and c.577 C > T; p.Pro193Ser in the *COQ4* gene, respectively. c.577 C > T variant in the *COQ4* gene has been reported in compound heterozygous form in two studies [11, 12].

**Table 2:** Annotations, minor allelic frequencies (MAF) and in silico prediction tools for the identified variants

Family ID	Gene	Transcript	Genomic position	cDNA position (HGVS)	Protein change (HGVS)	Type of variant	Zygosity	gnomAD	dbSNP ID	SIFT	CADD Score	Polyphen-2	ACMG	References
1	*CYP2U1*	ENST00000332884.10	*chr4-108866239 G > A*	*c.604G > A*	p.Glu202Lys	Missense	Homo	Exomes: ƒ =0.00000159 Genomes: NA	rs1733648064	Deleterious (0.01)	27.5	Probably damaging (0.999)	**LP** (PM2, PP3, PM3, PP5)	Zulfiqar et al. [9]
2	*ATXN1*	ENST00000244769.8	*Exon 8*	*ATXN1*(expanded): (CAG)46/(CAG)27	Normal range of CAG: < 35 repeats	Expansion Repeats	Het	NA	NA	NA	NA	NA	**P**	**Novel in terms of expanded CAG **
3	*ATM*	ENST00000452508.7	*chr11-108098533 C > T*	*c.103 C > T*	p. Arg35Ter	Stop gained	Homo	Exomes: ƒ=0.00000821 Genomes: ƒ = 0.0000132	rs55861249	NA	18.5	NA	**P** (PVS1, PM3, PM5)	Gilad et al. [10]
4	*COQ4*	ENST00000300452.8	*chr9-131095173 C > T*	*c.577 C > T*	p.Pro193Ser	Missense	Homo	Exomes: ƒ = 0.000000684 genomes: NA	rs568200779	Deleterious (0)	26.4	Probably damaging (0.999)	**LP** (PP3, PM2, PM3, PP5)	Reported in compound heterozygous form by Laugwitz et al. [11]; Mero et al. [12]

*LP,* Likely pathogenic; *P,* Pathogenic; *NA,* Not available

## Discussion

Spastic ataxia is characterized by the coexistence of cerebellar ataxia and spasticity, often accompanied by additional pyramidal signs. It represents a key feature of certain hereditary ataxias; however, it may also be observed in specific forms of hereditary spastic paraplegia as well as in various acquired neurological disorders [13]. Overlapping symptoms of these syndromes are challenging; it is essential to conduct an in-depth clinical evaluation and characterization of the inheritance and clinical course of the spastic ataxia to rule out the correct diagnosis.

Herein, we enrolled Pakistani families with a wide range of diverse phenotypes, including spastic paraplegia 56, autosomal recessive, OMIM: 615,030 (family 1), spinocerebellar ataxia Type 1, OMIM:164,400 (family 2), spastic paraplegia 26, ataxia telangiectasia, OMIM:208,900 (family 3), and spastic ataxia-10, autosomal recessive, OMIM: 620,666 (family 4). In this study, four known pathogenic variants were identified in *CYP2U1*, *ATXN1*, *ATM*, and *COQ4* in four unrelated families.

In family 1, we identified a previously reported missense (c.604G > A; p.Glu202Lys) homozygous mutation of the *CYP2U1* (NM_183075.3) gene located on chromosome 4 and associated with spastic paraplegia 56, autosomal recessive (OMIM: 615030), as confirmed by WES. To date, 52 mutations have been accessible in the Human Gene Mutation Database for the *CYP2U1* gene (HGMD 2023.1; http://www.hgmd.cf.ac.uk) [14]. In our family, two males are affected with a spastic paraplegia 56-associated phenotype, whereas the cited research study reports two females and one male with a similar phenotype [9]. Both affected individuals (IV: 2 & IV: 4) in our study had severe intellectual disabilities (IDs), while in the cited study, one male was mildly affected, and a female was severely affected in terms of ID. Furthermore, our patients were seizure-free, as was one of the three patients investigated by Zulfiqar et al. [9]. This comparison is variant-specific, and these observations highlight the variable expressivity of *CYP2U1*-associated disease and suggest that additional genetic, epigenetic, or environmental factors may influence disease severity and clinical presentation.

Spastic paraplegia 56 (SPG56) is an autosomal recessive-based, rare, early-onset, and complex type of hereditary spastic paraplegia (HSP) caused by *CYP2U1*. The candidate gene encodes a member of the cytochrome P450 family 2. The encoded protein is primarily expressed in the brain and thymus [15]. SPG56 was recently added to the HSPs list for the first time, as reported by Tesson et al. in 2012 [16]. 

Family 2 was diagnosed with spinocerebellar ataxia type 1, OMIM:164,400, having an autosomal dominant pattern of inheritance of the *ATXN1* gene located on chromosome 6p22.3 and caused by the expansion of the CAG trinucleotide repeats. We identified two alleles on the agarose gel: a normal (CAG)27 allele and an expanded (CAG)46 allele in the proband (II:2). His affected son (III:3) showed a normal (CAG)27 allele and an expanded (CAG)42 allele (Fig. 1).

SCA1 is clinically characterized by weak muscles and ataxia with difficulties in swallowing and speech. With time, the phenotypes progress to cognitive abnormalities, muscle atrophy, and respiratory deficiencies because of bulbar dysfunction, which is one of the leading causes of death [17]. Upon diagnosis, it mostly occurs in the third and fourth decades of life. The lifespan of this disease ranges from 10 to 30 years. Furthermore, the disease progression and onset depend on the number of trinucleotide repeats (CAG) expansions [18]. The normal CAG repeats in *ATXN1* are 6–35 CAG trinucleotides, and *ATXN1* alleles with more than 21 CAG repeats usually contain 1–3 interrupting CAT codons [19]. Expansion of one *ATXN1* allele with ≥ 39 uninterrupted CAG repeats, or ≥ 43 interrupted CAG repeats, causes late-onset, highly penetrant, progressive spinocerebellar ataxia. At the lower end of the expansion range, 39 uninterrupted CAG repeats are associated with the typical SCA1 phenotype, whereas interrupted 39-repeat alleles are not pathogenic. Alleles with 35 to 38 CAG repeats are associated with ataxia but generally lack the additional characteristic features of SCA1 [20]. 

Family 3 was diagnosed with ataxia-telangiectasia, OMIM:208,900, and carries a reported variant in the *ATM* gene (c.103 C > T; P. Arg35Ter). This detected variant (rs55861249) is a homozygous nonsense (stop-gained) variant classified as pathogenic by the ACMG guidelines. Gilad et al. [10] previously reported this variant in the *ATM* gene (NM_000051.4), matching our findings for ataxia-telangiectasia. His study was unique at that time because he demonstrated a founder effect in a large Jewish community [10]. 

In Family 4, *COQ4* (NM_016035.5) was mutated with matching symptoms of autosomal recessive spastic ataxia-10 (SPAX10). SPAX10 is a movement disorder with different ages of onset (infancy to adulthood) characterized by abnormal gait due to spasticity and lower limb hyperreflexia and/or limb ataxia and cerebellar gait. Furthermore, dysarthria, mild eye abnormalities, and cerebellar atrophy in some patients are the more variable features [21]. Lin et al. [22] diagnosed seven patients in China and Taiwan with SPAX10, with variable features such as hyperreflexia, walking difficulties, and extensor plantar responses, while one subject had seizures. Our variant is novel in terms of biallelic form, while a compound heterozygous (c.577 C > T (p.Pro193Ser) and c.718 C > T (p.Arg240Cys) pattern was reported in two different studies [11, 12]. Mero et al. [12] reported a single patient and observed generalized hypotonia, dysmetria, sialorrhea, and gait and axial ataxia with postural instability. The other notable symptoms were speech delay and cognitive impairment.

## Conclusion

The conducted research study highlights the need to prioritize previously known pathogenic variants and underscores the significance of previously identified loci. In addition, the usefulness of NGS or WES is explored as a convenient and efficient approach for genetically and clinically complicated neurodegenerative disorders. Through this approach, we can enhance genetic counseling, prenatal diagnosis, prognosis, and understanding of biological pathways for better therapeutic interventions.

## Limitations

In the current study, electrophysiological data and longitudinal therapeutic outcomes were not available for all affected individuals. Haplotype analysis, ancestry assessment, and population frequency comparison were not conducted in the study due to resource limitations. For Family 3 with ataxia-telangiectasia, additional laboratory parameters related to malignancy and immunodeficiency, including IgA/IgG levels and alpha-fetoprotein (AFP), were not performed due to resource constraints, representing a limitation of the current study.

## Data Availability

No datasets were generated or analysed during the current study.
